# Invasive Crayfish Threaten the Development of Submerged Macrophytes in Lake Restoration

**DOI:** 10.1371/journal.pone.0078579

**Published:** 2013-10-24

**Authors:** Jessica E. M. van der Wal, Martijn Dorenbosch, Anne K. Immers, Constanza Vidal Forteza, Jeroen J. M. Geurts, Edwin T. H. M. Peeters, Bram Koese, Elisabeth S. Bakker

**Affiliations:** 1 Department of Aquatic Ecology, Netherlands Institute of Ecology (NIOO-KNAW), Wageningen, The Netherlands; 2 Department of Aquatic Ecology and Environmental Biology, Radboud University Nijmegen, Nijmegen, The Netherlands; 3 Aquatic Ecology and Water Quality Management Group, Wageningen University, Wageningen, The Netherlands; 4 European Invertebrate Survey – The Netherlands, Leiden, The Netherlands; Smithsonian's National Zoological Park, United States of America

## Abstract

Submerged macrophytes enhance water transparency and aquatic biodiversity in shallow water ecosystems. Therefore, the return of submerged macrophytes is the target of many lake restoration projects. However, at present, north-western European aquatic ecosystems are increasingly invaded by omnivorous exotic crayfish. We hypothesize that invasive crayfish pose a novel constraint on the regeneration of submerged macrophytes in restored lakes and may jeopardize restoration efforts. We experimentally investigated whether the invasive crayfish (*Procambarus clarkii* Girard) affects submerged macrophyte development in a Dutch peat lake where these crayfish are expanding rapidly. Seemingly favourable abiotic conditions for macrophyte growth existed in two 0.5 ha lake enclosures, which provided shelter and reduced turbidity, and in one lake enclosure iron was added to reduce internal nutrient loading, but macrophytes did not emerge. We transplanted three submerged macrophyte species in a full factorial exclosure experiment, where we separated the effect of crayfish from large vertebrates using different mesh sizes combined with a caging treatment stocked with crayfish only. The three transplanted macrophytes grew rapidly when protected from grazing in both lake enclosures, demonstrating that abiotic conditions for growth were suitable. Crayfish strongly reduced biomass and survival of all three macrophyte species while waterfowl and fish had no additive effects. Gut contents showed that crayfish were mostly carnivorous, but also consumed macrophytes. We show that *P. clarkii* strongly inhibit macrophyte development once favourable abiotic conditions for macrophyte growth are restored. Therefore, expansion of invasive crayfish poses a novel threat to the restoration of shallow water bodies in north-western Europe. Prevention of introduction and spread of crayfish is urgent, as management of invasive crayfish populations is very difficult.

## Introduction

**Figure 1:**
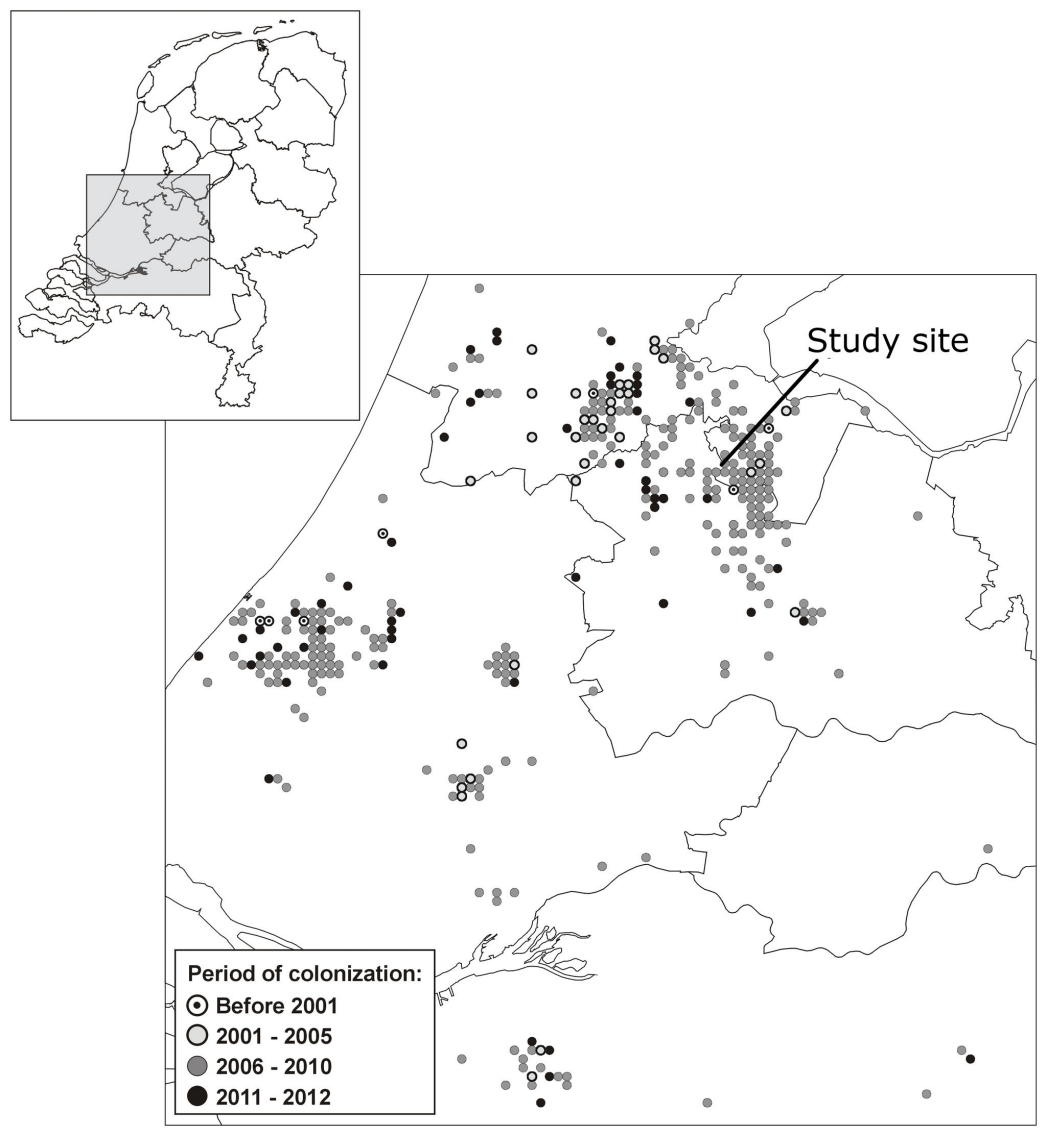
Map of records of the exotic crayfish *Procambarus clarkii* in the Netherlands. The data are a combination of (muskrat) trapping surveys, netting surveys and sightings of specimens migrating overland, n=1534 records. The study site is located at the lower tip of the black line.

Submerged macrophytes play a key role in shallow freshwater ecosystems by increasing nutrient retention, stabilizing sediment and providing food and habitat for macro-invertebrates, fish and birds [1]. A high abundance of submerged macrophytes is therefore considered to be an important variable in maintaining the clear water state in shallow lakes [2]. However, increased nutrient loading of shallow water systems during the last decades resulted in turbid waters and a strong decline of macrophyte abundance [3,4]. To restore water transparency and macrophyte vegetation, external nutrient loading has been reduced and additional measures like the removal of benthivorous fish have been taken [5-8]. These measures have only been temporarily successful [7]. Especially in lakes that are rich in organic sediments, internal phosphorus (P) loading still leads to high nutrient levels [9,10]. To minimize P release from lake sediments into the water column, several chemical phosphorus-binding agents have been applied, like calcium, aluminium and iron [11-13], leading to reduced internal P loading and increased water transparency in several studies [11,14]. However, increased water transparency does not always result in the return of submerged macrophytes [6,15]. This can be due to other unsuitable abiotic conditions for macrophyte development or to limiting biotic factors such as grazing by herbivores [16]. Waterfowl and fish can strongly reduce biomass of planted macrophytes in restored lakes [17-21] as well as spontaneous development of macrophyte communities [22,23], even though the latter is not found in all restoration projects [24-26]. However, large fish and waterfowl are no longer the only potential grazers as European shallow lakes are increasingly colonised by invasive crayfish such as the red swamp crayfish (*Procambarus clarkii*) [27-29]. In The Netherlands currently six species of exotic crayfish have established, whereas the native crayfish *Astacus astacus* is almost extinct due to the crayfish plague [30]. Crayfish may reduce the standing stock of macrophytes by direct consumption [31,32], increase water turbidity through sediment resuspension [33] and destroy macrophyte biomass by non-consumptive plant shredding [34], leading to a severe reduction of macrophyte abundance in lakes where they have been introduced [31,35-37]. Additionally, invasive crayfish may prevent the recruitment of macrophytes as shown in rice fields and mesocosm studies [38]. Therefore, invasive crayfish may potentially inhibit or prevent the return of macrophytes when abiotic conditions for macrophyte growth have been restored, but their impact in lake restoration projects remains untested. In The Netherlands, *P. clarkii* was first observed in 1985 [30] and has rapidly spread throughout the peat district in the west of the country in the last decade(Figure 1). Many restoration projects have been executed to restore the water transparency and promote the return of macrophytes in the shallow water bodies of this peat district [4,39,40]. 

We hypothesize that invasive crayfish pose a novel constraint on the regeneration of submerged macrophytes in lake restoration projects and may jeopardize restoration efforts.

The innovation of our study is that (1) we study the impact of crayfish in the field in an additive design, using different mesh size exclosures to study the role of crayfish versus other potential herbivores, and that (2) we study whether crayfish inhibit the return of macrophytes, when abiotic conditions for growth seem favourable. There has been documentation that water birds and large fish may jeopardize restoration efforts [17-23], but we are the first, to our knowledge, to show that invasive crayfish may also threaten successful lake restoration, e.g. the return of macrophytes. We show that invasive crayfish *P. clarkii* strongly inhibit macrophyte development once favourable abiotic conditions for macrophyte growth are restored. We conclude that invasive crayfish may compromise restoration measures and that the continuing expansion of invasive crayfish populations throughout north-western Europe poses a new threat to successful restoration of clear water with abundant submerged vegetation.

## Materials and Methods

### Ethics statement

The study was conducted on the terrain of Waternet. Waternet gave permission to work on their property as well as to conduct this study. No further permits were required for the described study, which complied with all relevant regulations. The study did not involve endangered or protected species.

### Study design

We experimentally tested the effect of the invasive crayfish *P. clarkii* on the development of submerged macrophytes within a restored shallow peat lake in The Netherlands. We used two enclosed lake sections, hereafter called ponds, where seemingly favourable abiotic conditions for macrophyte growth were found. In situ enclosures and exclosures in both ponds allowed us to investigate separate and combined effects of crayfish and native herbivores (fish and waterfowl) on the growth of three introduced plants. We analysed diet composition of *P. clarkii* using gut content analysis to determine whether they consumed the plants.

### Study area

**Figure 2:**
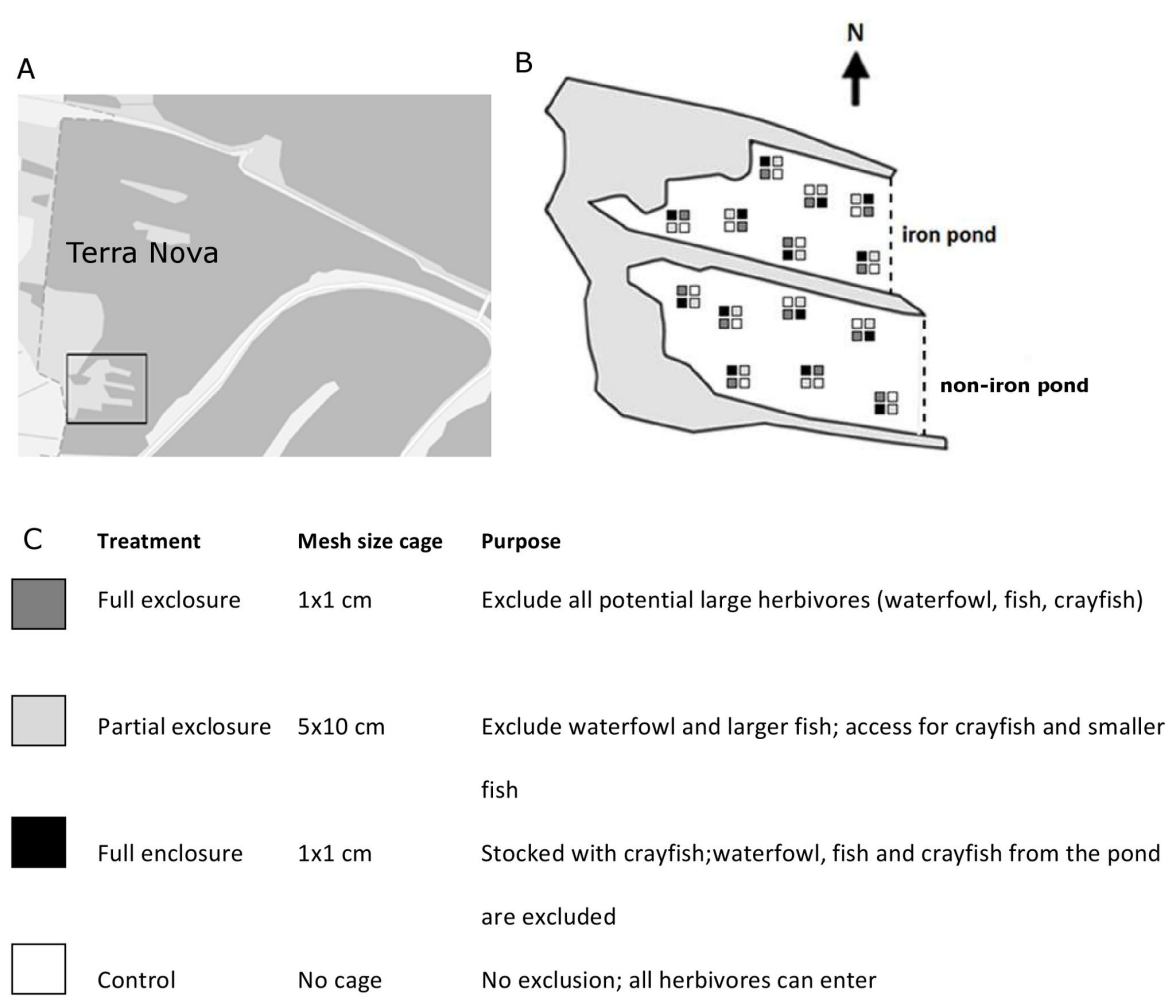
Overview of Lake Terra Nova and design of the cage-experiment. (A) Lake Terra Nova with ponds indicated in the black box. (B) Enlarged overview of the study ponds with the grazing treatments arranged in blocks within the iron pond (iron suppletion) and non-iron pond. (C) Legend of the grazing treatments applied. In the partial exclosure, mesh size was 5 cm height and 10 cm width to allow undisturbed access for large crayfish.

The experiment was conducted in the western part of Lake Terra Nova (52°13’N, 5°02’E), The Netherlands (Figure 2). Lake Terra Nova is an 85 ha shallow peat lake in which different restoration measures were taken in the past. The lake has a mean depth of 1.4 m and the bottom is covered with a 0.9 m organic sediment layer. Until the early 1970’s, a highly developed macrophyte community consisting of various Characeae and *Potamogeton* *sp.*, covered the lake bottom [21]. An increase in P loading was observed after 1977 and as a consequence the lake shifted from a clear macrophyte-dominated system to a turbid algae-dominated system in which only floating and sparse submerged macrophytes remained [21]. In 2003, biomanipulation was applied in which the benthivorous sediment disturbing fish assemblage was reduced from 180 kg ha^-1^ to less than 25 kg ha^-1^ cyrpinid fish biomass, which resulted in clear water and the return of many macrophyte species [21]. However, despite continued fishing keeping the cyprinid fish at low biomass, the macrophyte revival was only brief and in 2010 most of the lake contained bare sediment with scattered floating plant vegetation and turbid water through summer algal blooms. Red swamp crayfish were first reported in 2006 in the lake area (Figure 1) and may have been present since the early 2000’s, but numbers have not been documented. To test whether restoration measures would prevent algal blooms and stimulate the return of submerged macrophytes, two ponds of approximately 0.5 ha each were constructed in the western part of Lake Terra Nova in 2003 (Figure 2). In one pond FeCl_3_ was applied in 2009 to reduce internal P loading (gradual addition over a period of 102 days to a total of 85 g Fe m^-2^). However, in both ponds clear water conditions existed, whereas no submerged macrophytes were observed in either pond in 2009 or 2010 prior to this study and only floating leaved species (*Nuphar lutea* L. and *Nymphaea alba* L.) were present and *Phragmites australis* (Cav.) Trin. ex Steud. was the dominant species along the shores. We counted and sampled the potential herbivores, respectively water birds, fish and crayfish in and around the ponds (see Table 1 and 2 for methods, densities and species of waterbirds and fish). Crayfish abundance was determined by surveying both ponds simultaneously with 12 cylindrical crayfish traps (75 cm long, diameter 30 cm, 1.2 x 1.2 cm mesh) baited with cat food, which were checked every three days for five weeks prior to the experiment. Crayfish were individually marked. Only two crayfish were recaptured; numbers are therefore minimum number of crayfish present.

**Table 1:** Overview of densities of waterfowl around the ponds.

Waterfowl^a^	Individuals ha^-1^
Tufted duck (*Aythya fuligula* L.)	4.3
Eurasian coot (*Fulica atra* L.)	2.9
Common pochard (*Aythya farina* L.)	1.4
Greylag goose (*Anser anser* L.)	1.4
Gadwall (*Anas strepera* L.)	1.4
Egyptian goose (*Alopochen aegyptiacus* L.)	0.7
Mallard (*Anas platyrhynchos* L.)	0.7
Mute swan (*Cygnus olor* Gmelin)	0.6

a Water birds present in the water in and around the ponds (an area encompassing 0.07 km^2^) were counted weekly in April and May 2011 using binoculars, data are means of the weekly counts.

**Table 2:** Numbers of fish caught in the study ponds.

Fish CPUE^a^	Electro fishing (Individuals ha^-1^)			Gill nets (Individuals m^-1^ net)		
	Non-iron pond	Iron pond	Fish length range (cm)	Non-iron pond	Iron pond	Fish length range (cm)
Rudd (*Scardinius erythrophthalmus* L.)	35	69	3-7	0	0.008	14
Perch (*Perca fluviatilis* L.)	2482	414	7-15	0.16	0.24	8-22
Ruffe (*Gymnocephalus cernuus* L.)	0	0		0.03	0.008	7-13
Pike (*Esox Lucius* L.)	69	0	30-74	0	0	
Tench (*Tinca tinca* L.)	35	0	3	0.016	0	43-47
Roach (*Rutilus rutilus* Rafinesque)	2	0	4-6	0	0	

a Fish catch per unit effort. Fish abundance in each of the ponds was determined on 25 and 26 October 2011. Shoreline abundance was determined by electrofishing (200 volt, 5 amp, 290 m shore line length sampled per pond, 1 m transect width). Open water fish abundance was determined by overnight placement of multi-mesh gill nets (10-110 mm; total length 75 m) and an additional gillnet (140 mm; length 50 m) and additionally for 2 hours during the day on 25 October.

At the start and the end of the experiment we sampled environmental variables from the water column and sediment in both ponds; see Methods S1 for the methodology and Table S1 for the results.

### Experimental set-up

To analyse the effect of different herbivores on the development of macrophytes we performed an experiment in both ponds with four different grazing treatments: a full exclosure in which all studied herbivores were excluded, a partial exclosure providing access to crayfish and small fish, an enclosure, stocked with only crayfish, and a control where all herbivores had access to (Figure 2). Exclosures and enclosures consisted of cages of 1 m^3^ and were closed on all six sides, control plots were 1 m^2^. The corners of each cage were fixed with bamboo poles in the sediment and the control plots were marked with a pole. In each pond, each treatment was replicated seven times following a randomized block design (Figure 2); plots within a block were 2 m apart from each other. Each block of four treatments was placed randomly in the pond, but at least 15 m from the nearest other treatment block at the start of the growing season in 2011 (April 18^th^ 2011). Water depth in the cages ranged between 0.7 - 0.9 m; none of the cages was completely submerged and thus no algae were growing on the top, allowing maximum light availability inside the cage.

Since no submerged macrophytes were present in the ponds, three species of submerged macrophytes known to have occurred in Lake Terra Nova [21] were collected from nearby ponds and introduced. Two shoots of *Chara virgata* Kützing (mean DW 0.54 g ± 0.02 SE), *Elodea nuttallii* (Planch.) St. John (0.12 ± 0.01 g), and *Myriophyllum spicatum* L. (0.14 ± 0.02 g) were planted in separate square plastic pots (11 x 11 x 12 cm; one pot per species and two shoots per pot) filled with sediment originating from the pond where they were subsequently planted. Two replicate pots of the three species were randomly mounted on metal frames (50 x 50 cm). These frames, thus containing a total of 6 pots each (2 replicates x 3 species), were subsequently placed in each grazing treatment.

For the enclosure treatment, crayfish were caught with crayfish traps in Lake Terra Nova at about 500 m distance from the ponds. Crayfish were placed in the enclosures on the day of capture. At the start of the experiment, four adult crayfish were introduced in each enclosure (mean biomass per crayfish 37.4 g ± 2.0 SE, N_tot_ = 56, female:male ratio 1:1.7). The crayfish density in the enclosures (150 g m^-2^ wet wt) approached the higher densities estimated for Lake Terra Nova (up till 191 g m^-2^ wet wt, [41]). Crayfish densities vary widely in the field and are reported to range from 0.8-13 individuals m^-2^ in the meta-analysis of Matsuzaki et al. [38], who use 140 g m^-2^ as a high density in their own experiments. Gherardi and Acquistapace [37] report 4 and 8 individuals m^-2^ as natural densities in Italy, whereas Rodriguez-Villafane et al. [33] estimate a density of approximately 1 individual m^-2^ for a Spanish lake, although they indicate that this is probably an underestimation of the real density.

### Harvest

Six weeks later (May 31^st^ 2011), when the canopy-forming species *M. spicatum* and *E. nuttallii* had reached the water surface in a majority of the full exclosure plots, the plants were harvested. Macrophytes from all treatments were harvested and transported to the lab, rinsed with running fresh water, dried for 48 h at 60°C and weighed. Crayfish were collected from the enclosure cages and frozen at -20°C for gut analysis. 

### Crayfish diet

Crayfish gut content analysis was performed on 41 individuals in total from the enclosures from both ponds (22 from the iron pond and 19 from the non-iron pond) and 20 from the natural population in the ponds (10 per pond) caught outside the treatment blocks at the end of the experiment with the same traps used to estimate crayfish numbers (see Table 2). The crayfish were dissected and the stomach was removed from each individual and subsequently washed out to dilute the gut contents [42]. Food items (recorded as either present or absent in each specimen) were identified to the nearest recognizable taxonomic level with a dissecting microscope.

### Presence of plant propagules

To investigate whether the sediment of the ponds contained viable plant propagules, in total 25 L of the upper 5 cm of the sediment from three random locations in each pond was collected during the harvest of the transplants (on May 31^st^ 2011). The pooled sediment sample of each pond was taken to the lab and distributed over three 60 L aquaria, resulting in a ca. 3 cm sediment layer in each aquarium. Aquaria were subsequently filled with tap water (15 cm depth), and placed in a greenhouse at 20 °C under natural light conditions. Plants were allowed to emerge during 18 weeks after which all plants that had emerged were counted and identified to species level.

### Data analysis

Survival and biomass data of the plants were analysed using R version 2.15.0 [43]. Since survival of transplants followed a binomial distribution, effects of grazing treatment and pond on survival were analysed by fitting generalized linear mixed effect models with pond, grazing treatment and their interaction as fixed factors and treatment block and plant duplicate as random factors. The biomass of the plants (logarithmically transformed) was analysed by fitting general linear mixed effect models. Models were fitted with the lmer function in the lme4 package [44]. To determine effects of fixed factors a likelihood ratio test was used to compare models with and without the variable of interest [45]. Post-hoc comparisons of means were made based on Tukey contrasts available in the multcomp package. Assumptions of normality for general linear mixed models were checked by plotting residuals and performing a Shapiro test on residuals.

## Results

### Herbivore presence

The herbivores and omnivores present in and around the ponds were water birds, fish and crayfish (Table 1 and 2). With respect to crayfish, only *Procambarus clarkii* was caught in the ponds. In total 178 crayfish were caught in the non-iron pond and 66 in the iron pond, corresponding to respectively 0.42 and 0.16 CPUE (individuals per trapnight, based on 12 traps and 35 nights in each pond). Both ponds were characterized by low numbers of fish, predominantly existing of smaller sized perch, although the non-iron pond also harboured some larger individuals of pike and tench (Table 2). The biomass of benthivorous fish (rudd, ruffe, tench and roach) amounts to 0.2 kg ha^-1^ averaged over both ponds (based on CPUE of electrofishing, weight data not shown).

### Effect of herbivores on macrophyte development

**Figure 3:**
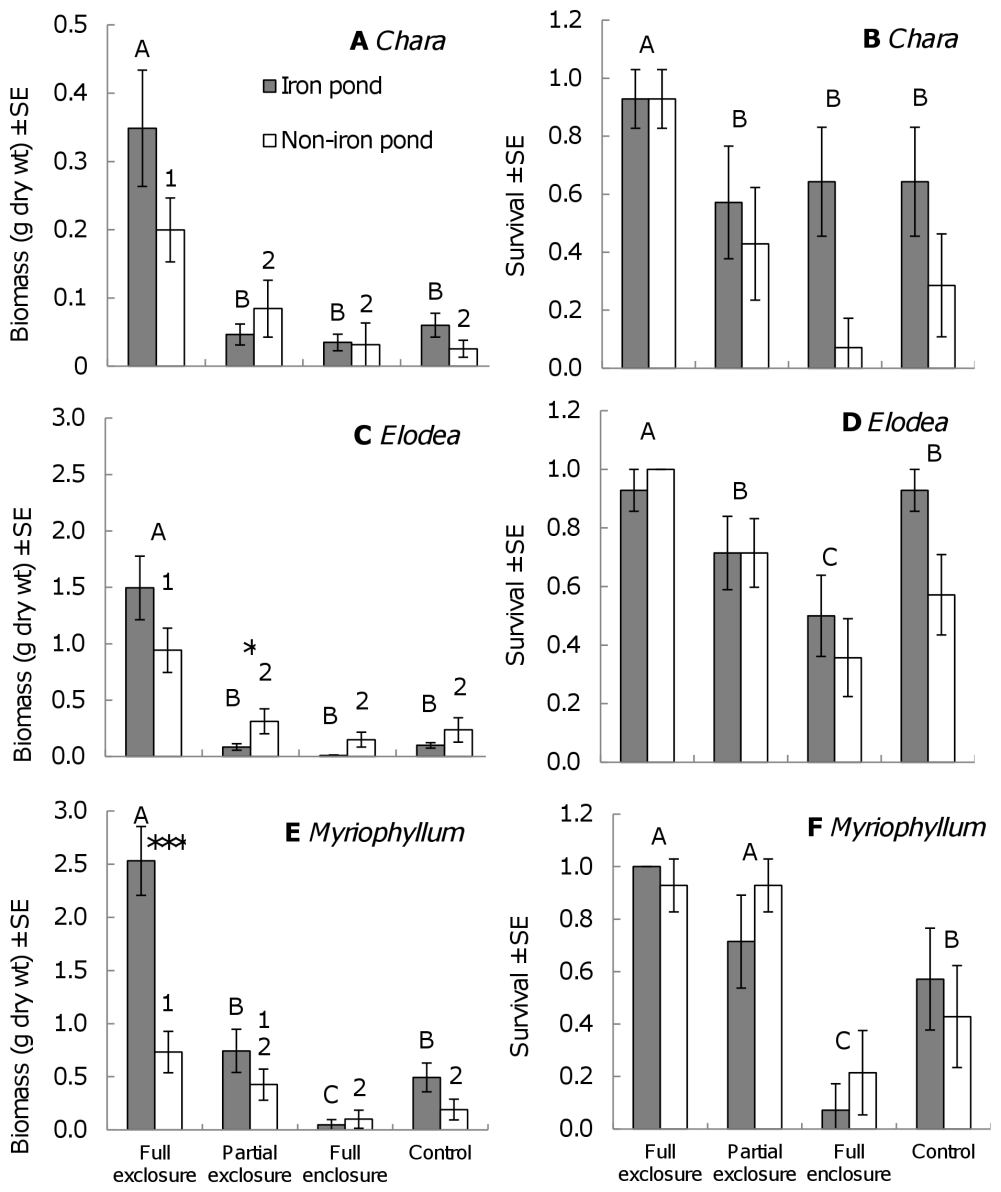
Biomass and survival of transplanted macrophytes under different grazing treatments. Mean biomass (left panels) and survival (right panels) of *C. virgata* (A,B), *E. nuttallii* (C,D), and *M. spicatum* (E,F) transplants at the end of the experiment for the non-iron and iron pond. Different letters or numbers in biomass panels indicate significant differences between treatments for the iron pond and non-iron pond respectively (Tukey post hoc comparisons, *P*<0.050). Significant differences in transplant biomass between ponds within a single treatment were found for *Elodea* biomass in the partial exclosure and for *Myriophyllum* biomass in the full exclosure and are indicated by asterisks (Tukey post-hoc comparisons, * *P*<0.050; ** *P*<0.01; ***P<0.001). For the survival panels, different letters indicate significant differences between treatments only. See Table 3 for results of the statistical analyses.

Macrophyte growth and survival was significantly affected by grazing treatment (Figure 3, Table 3). Free herbivore access strongly reduced survival and growth of all three macrophytes, which produced most biomass when fully protected from grazing (Figure 3, Table 3). Biomass of *E. nuttallii* and *C. virgata* was strongly reduced in all three treatments with herbivores. Similarly, biomass of *M. spicatum* was reduced in all treatments with herbivores in the iron pond, whereas in the non-iron pond, biomass in the partial exclosure was intermediate and not significantly different from the full exclosure or full enclosure and control (Figure 3, Table 3). The effect of grazing was stronger in the iron pond compared to the non-iron pond for *E. nuttallii* and *M. spicatum*. Biomass of *M. spicatum* was significantly higher in the full exclosures in the iron pond compared to the non-iron pond, whereas there was a similar trend, but no statistical differences, for *E. nuttallii* and *C. virgata* (Figure 3, Table 3). Survival of the macrophytes was similar in both ponds (Figure 3, Table 3). There was some mortality of crayfish in the enclosures, which had reduced stocked crayfish biomass in the enclosures whereas the surviving crayfish were growing, resulting in a final mean biomass per enclosure of 151.1 g ± 17.7 SE in the iron pond, and 132.4 g ± 9.4 in the non-iron pond which was not significantly different (t-test, df=12, t =0.932, *P*=0.370).

**Table 3:** Effect of pond (iron and non-iron) and grazing treatment on biomass and survival of three transplanted macrophyte species.

		Pond			Grazing treatment			Pond x Grazing		
Parameter	Species	Χ^2^	df	*P*	Χ^2^	df	*P*	Χ^2^	df	*P*
Biomass	*E. nuttallii*	11.56	4	0.021	95.77	6	<0.001	11.21	3	0.011
	*M. spicatum*	33.60	4	<0.001	91.84	6	<0.001	25.29	3	<0.001
	*C. virgata*	6.77	4	0.149	51.40	5	<0.001	5.55	3	0.136
Survival	*E. nuttallii*	7.17	4	0.127	27.74	6	<0.001	5.24	3	0.155
	*M. spicatum*	55.78	4	0.233	58.88	6	<0.001	5.32	3	0.150
	*C. virgata*	16.61	4	0.002	34.17	6	<0.001	5.42	3	0.143

Results (likelihood ratio tests) of general linear mixed effects models (biomass) and generalized linear mixed effect models (survival) per macrophyte species (see also Figure 3). Df – degrees of freedom.

### Germination of propagules

Each plot was checked for naturally emerging macrophytes in the field, but none were found on 31 May, after 6 weeks of exclosure treatments. Germination in the greenhouse showed that the sediment of both ponds contained viable propagules of macrophytes. Forty-eight individual macrophytes germinated from the sediment of both ponds combined, representing 8 species. In the sediment from the non-iron pond we found *Chara globularis* (3 individuals), *Myriophyllum spicatum* (4) and *Tolypella prolifera* (1), in the iron pond *Potamogeton pusillus* L. (1) as submerged species. *Nuphar lutea* (L.) Sm. was the only floating species and was found in both ponds (5 individuals in total). The emergent species were more abundant: *Typha angustifolia* L. (19), *Juncus articulatus* L. (4) and *Lythrum salicaria* L. (7), all species found in both ponds.

### Crayfish diet

Gut content analysis of the crayfish in the enclosures showed that the percentage of crayfish with animal remains in their stomach was considerably larger than the percentage of crayfish with vegetal remains in their stomach, whereas the majority of the free-living crayfish in the ponds had both animal as well as vegetal remains in their stomach (Table 4).

**Table 4:** Occurrence of food items (presence – absence) in crayfish guts from individuals collected from the full enclosures (n=41) and the natural population in the field (n=20), at the end of the experiment.

Identified food item:	Crayfish in enclosures	Free-living crayfish
Detritus	44	70
Remains of higher plants	17	75
Remains of filamentous algae	20	50
Diptera larvae	51	10
Crustacea	51	30
Gastropoda	5	10
Hydrachnidia	10	20
Protozoa - Amoeba	7	45
Unknown animal remains	22	55
Unknown remains	44	0
Subtotals:		
Animal remains	66	80
Vegetal remains	34	85

Data show the percentage of crayfish (in relation to the total number of dissected individuals) for which the given food item was present in the stomach.

### Environmental conditions

The abiotic conditions were very similar in both ponds (see Table S1). The iron pond had a higher attenuation of light, despite lower chlorophyll-a concentration, but in both ponds there was on average more than 15% of ambient light available at the bottom. The iron pond had a significantly higher Fe concentration in the surface water and sediment and a higher sediment P concentration. P and PO_4_ in the water column were higher at the start but lower at the end of the experiment, whereas NO_3_ was lower at the start and higher at the end in the iron pond compared to the non-iron pond respectively (Table S1).

## Discussion

Invasive crayfish *P. clarkii* can inhibit the development (growth and survival) of submerged macrophytes while abiotic conditions for macrophyte growth were favourable as demonstrated in our experiment. Survival and biomass of the three submerged macrophytes was significantly lower when crayfish were present, whereas the plant species grew well in both study ponds when they were protected from crayfish and other herbivores. When protected from grazing, *Myriophyllum* grew better in the iron pond, but there was no significant difference for the other species. The establishment of the ponds as lake enclosures may have provided enough shelter from the wind to prevent sediment resuspension and allow clear water conditions [21] regardless of iron addition, whereas differences among the ponds may have been present before the iron addition as well. We conclude that in both ponds, the light availability was with more than 15% of ambient light on the lake bottom (and often much more) above the minimum light requirements for growth of caulescent submerged angiosperms and charophytes [46] and therefore abiotic conditions were suitable for macrophyte growth in both ponds. During our experiment, we did not observe naturally emerging vegetation, which may perhaps be due to the short term (6 weeks) or early season (April-May) in which we performed the experiment. The presence of viable propagules of several submerged species in the sediment suggests that the absence of submerged vegetation in the entire ponds is not due to a lack of propagules per se. We therefore further focus on the role of invasive crayfish and their potential to inhibit macrophyte growth and development once favourable abiotic conditions for growth have been created.

Whereas invasive crayfish are known to reduce macrophyte abundance in southern and northern Europe [33,37,47] and inhibit propagule establishment in mesocosms [38], their impact on macrophyte establishment in field restoration projects has not yet been tested to our knowledge. We show that invasive crayfish may present a new bottleneck for macrophyte development in north-western European waters when abiotic conditions for macrophyte growth are restored. In north-western Europe, many lake restoration projects have been executed and are still being implemented, aimed at improving water transparency and development of abundant macrophyte vegetation [5-8,48]. Our results suggest that these projects may face a new constraint with the increasing spread of invasive crayfish, particularly *P. clarkii*. 

### Effects of crayfish versus other potential herbivores

The enclosure treatments with only crayfish present showed that crayfish strongly reduced survival and growth of submerged macrophytes. Furthermore, the very small differences between the enclosure treatment (access for crayfish only) and the partial exclosure (access for crayfish and small fish) and the control treatment (access for all herbivores) indicate strong effects of crayfish and no significant additive effects of waterfowl and larger fish. Smaller fish that could enter the partial exclosures were present in the study ponds. Technically, very small fish could even have entered the full exclosure or crayfish enclosure with the mesh size of 1 x 1 cm and reduce plant growth. However, this would have led to reduced growth of the macrophytes in the full exclosure, whereas we observed a much higher plant growth in the full exclosure compared to the treatments where larger herbivores had access. Therefore, if very small fish did enter the full exclosure, we estimate their impact on plant growth to be very small. Small fish may have entered the partial exclosure, in which the mesh was oriented such that it was 10 cm wide and 5 cm in height (to allow optimal access for large crayfish, which are wider than tall due to their claws). However, the density of fish in the study ponds was generally very low and most fish were not herbivorous. Of the fish that include macrophytes in their diet, e.g. rudd and tench, the smaller size classes are mostly carnivorous [49,50] and even the large fish of these species preferentially feed on macrofauna under temperate conditions, as demonstrated for rudd [51,52]. When feeding on invertebrates, fish may inadvertently ingest the macrophyte leaves which have macrofauna on them. Smaller roach (of 7 cm and larger) for instance have been observed to pluck macrophyte leaves when consuming macro-invertebrates on the leaves, although they mostly do so when zooplankton and other food sources are scarce [53]. This is in line with observations in a Finnish lake, where in spring, when zooplankton is abundant, small (<10 cm) rudd does not ingest plant material and only larger rudd consumed plants [50]. Furthermore, significant effects of plant plucking on macrophyte growth were observed in Lake Müggelsee at a fish biomass of >150 kg ha^-1^ of which 70-80% consisted of bream and roach [23,53]. In contrast, fish density in our study ponds was much lower with 0.2 kg ha^-1^ for benthivorous fish, estimated from the electrofishing CPUE. Previous removal of benthivorous fish in our study lake showed that a reduction from 180 to <25 kg ha^-1^ biomass of cyprinid fish, resulted in strong growth of submerged macrophytes [21]. Therefore, whereas we cannot entirely exclude that small fish may have had an additional impact on macrophyte growth in our study, a large part of the difference in plant growth among the partial exclosure and crayfish enclosure versus the full exclosure is likely caused by crayfish considering the low density and diet preferences of small fish and the high crayfish density. Whereas it was known that grazing by water birds or fish can be a limiting factor in the appearance of submerged vegetation [17-23], we now show that the presence of crayfish can inhibit the establishment of submerged macrophytes in a lake restoration project. The absence of an additional effect of water birds and large fish demonstrates that crayfish alone are potentially able to prevent restoration of submerged vegetation. 

### Crayfish grazing versus bioturbation

It is often unclear whether observed crayfish impact on macrophytes is caused by herbivory or bioturbation [38]. In our study, gut content analysis showed that *P. clarkii* had an omnivorous diet, with animal and plant material and detritus found equally often in free living crayfish. The gut of the crayfish in the enclosures contained more frequently animal material. This may be due to the fact that most plant material had already been consumed at the end of the experiment and thus was no longer available. These results agree with previous studies that showed crayfish to be omnivorous [28,47,53,54]. In our study the crayfish did consume macrophytes and thus at least part of their impact on macrophytes was due to herbivory. However, we cannot exclude that part of the observed effects of crayfish may also be due to bioturbation, particularly destruction or uprooting of the planted macrophytes [55].

### Effect of crayfish during lake restoration

Invasive crayfish may reduce macrophyte abundance and induce a shift to a turbid, algae dominated, state of the ecosystem [27,33]. The goal of many restoration projects is to reverse a turbid state into a clear water state dominated by submerged macrophytes [2]. Once appropriate measures have been taken macrophytes may return, when propagules are available [15,16,48]. The question is to what extent invasive crayfish may inhibit the return of submerged macrophytes and therefore compromise restoration efforts. The impact of crayfish on the establishment and development of submerged macrophytes is potentially large as they live on the sediment, which is where macrophytes emerge from propagules. Crayfish have been shown to strongly suppress macrophyte establishment from a propagule bank in mesocosm studies [38]. Contrary to herbivorous waterfowl, which are frequently mentioned as consumers of establishing macrophytes [18-20], crayfish stay in a lake year round and are able to feed on alternative sources like detritus [56] on which they can sustain themselves when macrophytes are absent [57]. As a result, crayfish density will not be strongly coupled to the availability of macrophytes in lakes with organic sediments, such as our study lake. Therefore, grazing pressure on macrophytes is potentially high, particularly when predation on the crayfish is low, for instance when fish densities are low due to biomanipulation, as is the case in our study lake [40].

Species invasions in general occur more often in disturbed situations [58] where exotic species can opportunistically invade (temporarily) empty niches [59]. *P. clarkii* is an opportunistic species due to its omnivorous feeding habits and semi-amphibious life style [28,57]. Possibly lake restoration projects are more prone to colonization by invasive crayfish, but to our knowledge, this has not been investigated.

## Conclusions

We conclude that *P. clarkii* strongly reduced the biomass development and survival of establishing macrophytes. Invasive crayfish may form a new constraint on the development of submerged aquatic vegetation when abiotic conditions for macrophyte growth are improved. Invasive crayfish may compromise restoration measures and pose a new threat to successful restoration of clear water with abundant submerged vegetation. The continuing expansion of invasive crayfish populations throughout north-western Europe is worrying. Strong emphasis should be put on prevention of introduction and where possible spread of the crayfish, since removal or management of invasive crayfish populations is very difficult [32]. 

## Supporting Information

Methods S1 **Collection of environmental and chemical variables.**
(DOCX)

Table S1 **Abiotic characteristics of surface water, pore water, and the sediment of the two experimental ponds (iron and non-iron).**
(DOCX)
