# Supplementary material for: Invasive Crayfish Threaten the Development of Submerged Macrophytes in Lake Restoration
Source: PLoS One. 2013 Oct 24;8(10):e78579. doi: 10.1371/journal.pone.0078579 (PMC3813481; doi:10.1371/journal.pone.0078579)
Supplement: Methods S1 — Collection of environmental and chemical variables. (DOCX) [file pone.0078579.s001.docx]

**Supporting Information** belonging with van der Wal et al. “Invasive crayfish threaten the development of submerged macrophytes in lake restoration”

**Methods S1. Collection of environmental and chemical variables**

Environmental and chemical variables were measured in both ponds at the start and the end of the experiment (Table S1). Surface water samples from 10 cm below the water surface were collected in 500 mL polyethylene bottles. Sediment pore water was collected anaerobically using 60 mL vacuum syringes connected to ceramic soil moisture samplers (Eijkelkamp Agrisearch Equipment, Giesbeek, the Netherlands), which were installed in the upper 10 cm of the sediment. The first 10 mL was discarded to enable anaerobic sampling. The pH of the water samples was measured using a combined pH electrode with an Ag/AgCl internal reference (Orion Research, Beverly, CA, USA), and a TIM800 pH meter. Subsequently, surface water samples were filtered through glass microfiber filters (type GF/C, Whatman, Brentford, UK). The samples were stored in polyethylene bottles at -20 °C until further analyses.

Additionally to nutrients, water transparency (by absorption at 750 nm in a Helios delta photospectrometer, Unicam, Cambridge, UK) and chlorophyll-a concentration (in a PhytoPAM phytoplankton Analyser, Heinz Walz GmbH, Effeltrich, Germany), of the surface water was measured in 100 ml water samples (replicated three times), whereas light extinction in the water column was measured at a depth of 60 cm (by a LI-CORLI-250 quantum photometer, LI-COR Biosciences, Lincoln, NE, USA, replicated seven times).

Nutrients (Fe, S, P, organic P and Olsen-P) in the pond sediments were only measured prior to the transplant experiment. Samples of the upper sediment layer were taken with a multisampler (Eijkelkamp Agrisearch Equipment, Giesbeek, The Netherlands), transported in airtight bags and kept in the dark at 4°C until further analyses.

Homogenized portions of 5 g wet sediment were used to determine organic P concentrations using a P-fractionation analysis according to Golterman [1]. The rest of the sediment was dried for 48h at 70°C. Homogenized portions of 3 g dry sediment were used to determine Olsen-P concentrations by extraction according to Olsen et al. [2]. Homogenized portions of 200 mg dry sediment were digested with 4 mL HNO_3_ (65%) and 1 mL H_2_O_2_ (30%), using an Ethos D microwave labstation (Milestone srl, Sorisole, Italy). Digestates were diluted and concentrations of Fe, S, and P were determined by ICP (see below).

The concentrations of PO_4_, NO_3_, and NH_4_ in surface water and sediment pore water were measured colorimetrically with an Auto Analyser 3 system (Bran+Luebbe, Norderstedt, Germany) according to Geurts et al. [3]. The concentrations of Fe, S, P, organic P, and Olsen-P were measured using an ICP Spectrometer (IRIS Intrepid II, Thermo Electron Corporation, Franklin, USA).

Differences in abiotic characteristics were analysed using general linear mixed effect models (see methods in main manuscript). Pond, sampling time and their interaction were fixed factors. The factor sampling time was defined as a random slope and nested in the random factor sampling location to account for repeated measurement correlations.

**References**

1. Golterman HL (1996) Fractionation of sediment phosphate with chelating compounds: a simplification, and comparison with other methods. Hydrobiologia 335: 87-95.

2. Olsen SR, Cole CV, Watanabe FS, Dean LA (1954) Estimation of available phosphorus in soils by extraction with sodium bicarbonate. Vol. Cir. No 939, U.S. Dept. of Agriculture, Washington DC, pp. 1-19.

3. Geurts JJM, Smolders AJP, Verhoeven JTA, Roelofs JGM, Lamers LPM (2008) Sediment Fe:PO_4_ ratio as a diagnostic and prognostic tool for the restoration of macrophyte biodiversity in fen waters. Freshwater Biol. 53: 2101-2116.
