# Supplementary material for: Invasive Crayfish Threaten the Development of Submerged Macrophytes in Lake Restoration
Source: PLoS One. 2013 Oct 24;8(10):e78579. doi: 10.1371/journal.pone.0078579 (PMC3813481; doi:10.1371/journal.pone.0078579)
Supplement: Table S1 — Abiotic characteristics of surface water, pore water, and the sediment of the two experimental ponds (iron and non-iron). (DOCX) [file pone.0078579.s002.docx]

**Supporting Information** belonging with van der Wal et al. “Invasive crayfish threaten the development of submerged macrophytes in lake restoration”

**Table S1.** Abiotic characteristics (mean ± SE) of surface water, pore water, and the sediment of the two experimental ponds (iron and non-iron).

| Place of sampling | Variable | n | Pond | At the start | | At the end (6 weeks later) | | Model results | | |
| --- | --- | --- | --- | --- | --- | --- | --- | --- | --- | --- |
| Surface water |  |  |  | Mean | SE | Mean | SE | Pond | Time | Pond x Time |
|  | pH | 4 | Iron | 8.25 | 0.49 | 7.19 | 0.02 | NS | *** | NS |
|  |  | 4 | Non-iron | 8.46 | 0.02 | 7.20 | 0.02 |  |  |  |
|  | Fe (µmol L^-1^) | 4 | Iron | 18.71 | 3.93 | 28.75 | 3.44 | *** | * | NS |
|  |  | 4 | Non-iron | 2.42 | 0.04 | 4.30 | 0.37 |  |  |  |
|  | P (µmol L^-1^) | 4 | Iron | 1.81 | 0.09 | 1.24 | 0.09 | ** | ** | *** |
|  |  | 4 | Non-iron | 1.22 | 0.04 | 1.50 | 0.16 |  |  |  |
|  | S (µmol L^-1^) | 4 | Iron | 94.13 | 2.74 | 49.68 | 8.35 | NS | *** | NS |
|  |  | 4 | Non-iron | 98.14 | 0.29 | 48.00 | 1.15 |  |  |  |
|  | PO_4_ (µmol L^-1^) | 4 | Iron | 0.54 | 0.13 | 0.23 | 0.05 | * | * | ** |
|  |  | 4 | Non-iron | 0.26 | 0.02 | 0.45 | 0.10 |  |  |  |
|  | NO_3_ (µmol L^-1^) | 4 | Iron | 3.85 | 3.28 | 7.07 | 4.15 | ** | *** | *** |
|  |  | 4 | Non-iron | 16.52 | 0.68 | 0.00 | 0.00 |  |  |  |
|  | NH_4_ (µmol L^-1^) | 4 | Iron | 4.85 | 2.44 | 14.91 | 13.11 | NS | NS | NS |
|  |  | 4 | Non-iron | 5.57 | 1.24 | 28.08 | 15.61 |  |  |  |
|  | Chlorophyll-a (µg L^-1^) | 3 | Iron | 0.74 | 0.07 | 0.80 | 0.13 | ** | NS | NS |
|  |  | 3 | Non-iron | 1.33 | 0.17 | 5.40 | 0.14 |  |  |  |
|  | Light extinction (%) | 7 | Iron | 61.52 | 2.43 | 84.82 | 0.73 | *** | *** | *** |
|  |  | 7 | Non-iron | 52.47 | 2.20 | 55.63 | 2.17 |  |  |  |
|  | Water transparancy (750 nm) | 3 | Iron | 0.02 | 0.00 | 0.02 | 0.00 | NS | NS | NS |
|  |  | 3 | Non-iron | 0.03 | 0.00 | 0.05 | 0.01 |  |  |  |
| Pore water |  |  |  |  |  |  |  |  |  |  |
|  | pH | 6 | Iron | 6.60 | 0.06 | 6.57 | 0.03 | * | NS | NS |
|  |  | 6 | Non-iron | 6.70 | 0.02 | 6.77 | 0.07 |  |  |  |
|  | Fe (µmol L^-1^) | 6 | Iron | 92.43 | 18.75 | 102.20 | 17.01 | NS | NS | NS |
|  |  | 6 | Non-iron | 83.60 | 11.38 | 71.15 | 13.94 |  |  |  |
|  | P (µmol L^-1^) | 6 | Iron | 39.37 | 12.35 | 44.95 | 12.81 | NS | NS | NS |
|  |  | 6 | Non-iron | 28.09 | 4.84 | 26.43 | 6.13 |  |  |  |
|  | S (µmol L^-1^) | 6 | Iron | 25.78 | 7.03 | 18.49 | 0.64 | * | NS | NS |
|  |  | 6 | Non-iron | 14.34 | 1.01 | 14.46 | 1.05 |  |  |  |
|  | PO_4_ (µmol L^-1^) | 6 | Iron | 10.46 | 4.47 | 6.38 | 1.70 | NS | NS | NS |
|  |  | 6 | Non-iron | 5.65 | 2.16 | 4.32 | 1.36 |  |  |  |
|  | NO_3_ (µmol L^-1^) | 6 | Iron | 1.50 | 0.44 | 2.66 | 1.17 | NS | NS | NS |
|  |  | 6 | Non-iron | 1.18 | 0.13 | 0.44 | 0.15 |  |  |  |
|  | NH_4_ (µmol L^-1^) | 6 | Iron | 1011.80 | 376.79 | 1081.04 | 354.92 | NS | NS | NS |
|  |  | 6 | Non-iron | 878.16 | 146.79 | 810.22 | 170.75 |  |  |  |
| Sediment |  |  |  |  |  |  |  |  |  |  |
|  | Fe (µmol L^-1^) | 6 | Iron | 415.59 | 36.52 |  |  | ** |  |  |
|  |  | 6 | Non-iron | 271.67 | 13.03 |  |  |  |  |  |
|  | P (µmol L^-1^) | 6 | Iron | 37.59 | 2.33 |  |  | * |  |  |
|  |  | 6 | Non-iron | 31.13 | 1.32 |  |  |  |  |  |
|  | S (µmol L^-1^) | 6 | Iron | 512.19 | 28.82 |  |  | NS |  |  |
|  |  | 6 | Non-iron | 485.80 | 20.90 |  |  |  |  |  |
|  | Organic-P (µmol L^-1^) | 6 | Iron | 16.75 | 1.95 |  |  | NS |  |  |
|  |  | 6 | Non-iron | 17.79 | 2.13 |  |  |  |  |  |
|  | P-Olsen (µmol L^-1^) | 6 | Iron | 9.61 | 1.06 |  |  | *** |  |  |
|  |  | 6 | Non-iron | 3.52 | 0.98 |  |  |  |  |  |

For each variable, results (likelihood ratio tests) of general linear mixed models are reported in the columns 'pond', 'time', and 'pond x time'. Sediment variables were only collected once. NS: *P*>0.050; * *P*<0.050; ** *P*<0.010; *** *P*<0.001, n= number of samples taken.
